# In Vivo Assessment of Antioxidant Potential of Human Milk Treated by Holder Pasteurization or High Hydrostatic Pressure Processing: A Preliminary Study on Intestinal and Hepatic Markers in Adult Mice

**DOI:** 10.3390/antiox11061091

**Published:** 2022-05-31

**Authors:** Eve Wemelle, Lucie Marousez, Jean Lesage, Marie De Lamballerie, Claude Knauf, Lionel Carneiro

**Affiliations:** 1 INSERM U1220, Institut de Recherche en Santé Digestive (IRSD), Université Paul Sabatier, Toulouse III, CHU Purpan, Place du Docteur Baylac, CS 60039, CEDEX 3, 31024 Toulouse, France; eve.wemelle@inserm.fr (E.W.); lionel.carneiro@inserm.fr (L.C.); 2 European Associated Laboratory (EAL) «NeuroMicrobiota», International Research Projects (IRP) INSERM, 31024 Toulouse, France; 3 Inserm, CHU Lille, U1286-INFINITE-Institute for Translational Research in Inflammation, University of Lille, 59000 Lille, France; lucie.marousez@univ-lille.fr; 4 UMR CNRS 6144 GEPEA (Génie des Procédés Environnement-Agroalimentaire), ONIRIS, CS 82225, 44322 Nantes, France; marie.de-lamballerie@oniris-nantes.fr

**Keywords:** human milk, high hydrostatic pressure, holder pasteurization, H_2_O_2_, antioxidants, oxidative stress, inflammation, gut, liver

## Abstract

Preterm infants are highly susceptible to oxidative stress due to an imbalance between endogenous oxidant and antioxidant systems. In addition, these newborns are frequently fed with donor milk (DM) treated by Holder pasteurization (HoP) at 62.5 °C for 30 min, which is known to alter numerous heat-sensitive factors, including some antioxidants. High hydrostatic pressure (HHP) processing was recently proposed as an innovative method for the treatment of DM. The present study aimed to measure the redox balance of HoP- and HHP-DM and to study, in vivo, the effects of HoP- and HHP-DM on the gut and liver. H_2_O_2_, vitamin A and vitamin E (α- and γ-tocopherols) concentrations, as well as the total antioxidant capacity (TAC), were measured in raw-, HoP- and HHP-DM. The gene expression level of antioxidant systems and inflammatory response were quantified in the ileum and liver of adult mice after 7 days of oral administration of HoP- or HHP-DM. HoP reduced the γ-tocopherol level, whereas HHP treatment preserved all vitamins close to the raw milk level. The milk H_2_O_2_ content was reduced by HHP but not by HoP. The total antioxidant capacity of DM was reduced after HHP processing measured by PAOT-Liquid^®^ technology but was unaffected after measurement by ORAC assay. In mice, HHP-DM administration induced a stimulation of antioxidant defenses and reduced some inflammatory markers in both the ileum and liver compared to HoP-DM treatment. Our preliminary study suggests that the HHP processing of DM may better protect preterm infants from gut and liver pathologies compared to HoP, which is currently used in most human milk banks.

## 1. Introduction

During intrauterine life, the fetus is in a hypoxic environment with a partial pressure of oxygen of 20–25 mmHg. At birth, with the onset of respiration, the oxygen concentration doubles, leading to a high oxygen availability for tissues, but also to a rise in reactive oxygen species (ROS) production [1]. In preterm babies, this period is very critical, since these infants are highly susceptible to oxidative stress (OS). This OS is due to an imbalance between oxidant and antioxidant systems. Thus, preterm babies are at risk of “oxygen radical diseases” [1,2,3]. OS is an important factor responsible for the development of multiple pathologies in preterm newborns, such as necrotizing enterocolitis (NEC) [1,3,4]. NEC is the leading cause of death and disability from gastrointestinal disease in the premature infant. NEC affects between 1% and 12% of infants born before 37 weeks of gestation, and the risk is particularly high in those under 1.500 g of body weight [5,6]. The pathogenesis of NEC implies both an altered interaction between bacterial signaling receptors on the premature intestine and an abnormal gut microbiota that triggers a pro-inflammatory response in the intestinal mucosa [5]. In addition, during NEC, inflammatory cells release large amounts of ROS, resulting in cell damage, endothelial dysfunction and the overproduction of pro-inflammatory cytokines [5,7].

Clinical studies have shown that breast milk (as opposed to infant formula) reduces the NEC incidence in premature infants [8]. In hospitals, mothers of preterm infants are frequently unable to provide breast milk in sufficient amounts. Human milk banks (HMBs) provide donor milk (DM) as an alternative for the feeding of these preterm infants. In order to ensure the microbial safety of DM, most HMBs sterilize human milk using the standard method of Holder pasteurization (HoP), which is performed by heating milk to 62.5 °C for 30 min [9]. However, an increasing number of studies show that HoP degrades numerous important bioactive factors, such as immunoglobulins, lactoferrin, some vitamins, lysozyme, milk lipase and some hormones [9,10,11]. High hydrostatic pressure (HHP) may be an innovative method for the treatment of DM. Indeed, recent data have demonstrated that HHP maintains numerous bioactive factors, such as immunoglobulins, lactoferrin, lysozyme, milk lipase, oligosaccharides and several hormones at levels close to raw milk [10,11,12,13].

Human milk contains several antioxidant systems [14]. These antioxidant systems include enzymatic antioxidants and also numerous non-enzymatic antioxidants, such as glutathione and some vitamins (C, E and A), as well as trace elements, including zinc, copper and selenium. In addition, some short chain fatty acids and amino acids also have antioxidant properties [15,16]. Then, the preservation of milk compounds, including antioxidants, seems to be crucial for the optimal health of preterm newborns.

The present study aims to characterize the impact of the HoP and HHP treatment of DM on the concentration of some milk antioxidants, on the milk H_2_O_2_ level and on the total antioxidant capacity of DM. In addition, we evaluated for the first time in vivo the consequences of these treatments of DM. Our preliminary study was performed in adult mice subjected to a chronic oral administration (7 days) of HoP- or HHP-DM. The gene expression level of several intestinal and hepatic antioxidant enzymes, as well as intestinal and hepatic markers of inflammation, were quantified.

## 2. Materials and Methods

### 2.1. Milk Collection and HoP and HHP Processing

Frozen DM samples from 11 donors were provided by the regional HMB (Lactarium Régional de Lille, Jeanne de Flandre Children’s Hospital, CHU Lille). Donors provided written, informed consent for the use of their milk for this research purpose. After thawing of milk samples, 8 different batches of DM were created by mixing various volumes of all DM samples, primarily in order to homogenize breast milk composition. Three aliquots of DM were prepared for each batch: one fraction was stored at −80 °C without any other treatment (raw milk sample (RM)); one fraction was subjected to HoP according to the standard pasteurization protocol (62.5 °C for 30min); the last fraction was subjected to HHP processing as previously described [12]. The set of HHP parameters was as follows: pressure = 350 MPa, temperature = 38 °C, VA (application rate) = 1 MPa.s^−1^, number of cycles = 4 cycles, duration of each cycle = 5 min. Samples were stored at −80 °C until analysis.

### 2.2. Quantification of H_2_O_2_ and Antioxidants in Milk Samples

Vitamin A and vitamin E (α- and γ-tocopherols) determination was performed by high performance liquid chromatography (HPLC) (Alliance, Waters, Milford, MA, USA) coupled to a diode array detector (DAD) (PDA 2996, Alliance, Waters, Milford, MA, USA) using Chromsystems reagent (34,000, Chromsystems, Munich, Germany). Milk total antioxidant capacity (TAC) was determined using the electrochemical PAOT-Liquid^®^ Technology as previously described [17]. Briefly, 20 µL of milk samples was added to a reaction medium (1 mL physiological solution at pH 6.7–7.2, temperature 24–27 °C) containing a molecule in a free radical state called mediator (M^.^). Using two microelectrodes immersed in the medium, PAOT-liquid^®^ activity was estimated by recording electrochemical potential modifications in the reaction medium. PAOT-liquid^®^ activity is expressed as mg gallic acid equivalents (GAE) per liter. Milk TAC was also measured using an ORAC (oxygen radical absorbance capacity) assay in which there is a competitive reaction between the antioxidant and the substrate for the free radicals [18]. H_2_O_2_ concentration was measured in RM-, HoP- and HHP-DM. Spontaneous H_2_O_2_ release was measured at room temperature for 10 min by using a H_2_O_2_-specific amperometric probe (ISO-HPO, World Precision Instruments) directly immersed in the milk. The concentration of H_2_O_2_ in milk was measured in real-time (TBR1025, World Precision Instruments, Sarasota, FL, USA).

### 2.3. Mice

Nine-week-old male C57BL/6J mice (Charles River Laboratory, l’Arbresle, France) were housed in controlled environment (room temperature of 23 ± 2 °C, 12 h’ day-light cycle). Food and water were proposed ad libitum. Oral gavage of HoP- or HHP-DM (100 μL/day) was performed during 7 days. Mice were sacrificed under fed conditions. Ileum and liver were collected, washed and frozen at −80 °C until RT-qPCR experiments. All in vivo experiments were conducted according to the European Community regulations concerning the protection of experimental animals and were approved by the local Animal Care and Use Committee under the protocol number 2021042609281581.

### 2.4. Gene Expression

Homogenization of tissues, total RNA extraction, reverse transcription and real-time PCR were performed as previously described [19]. The sequences of primers used in this study are presented in Table 1. Quantification of gene expression was performed using the comparative Ct (threshold cycle) method, and data were normalized to HPRT (Hypoxanthine-guanine phosphoribosyltransferase) expression.

### 2.5. Statistics

Results are presented as mean ± SEM. Outliers were detected via a Grubb’s test. A D’Agostino–Pearson test was used to evaluate the normality. Statistical differences were tested by paired *t*-test or one-way ANOVA test. *p* ≤ 0.05 was considered as significant.

## 3. Results

### 3.1. Concentrations of Antioxidants, H_2_O_2_ Levels and Total Antioxidant Capacities of Raw DM and HoP- and HHP-DM

#### 3.1.1. Concentrations of Antioxidants

Vitamin A and α-tocopherol DM concentrations were not significantly modulated by HoP and HHP treatment (Table 2). γ-tocopherol levels were not different between raw- and HHP-DM. Conversely, HoP treatment significantly reduced γ-tocopherol concentrations compared to raw DM (−12%, *p* < 0.05).

#### 3.1.2. H_2_O_2_ Concentrations and Total Antioxidant Capacities of Raw-, HoP- and HHP-DM

**Figure 1:**
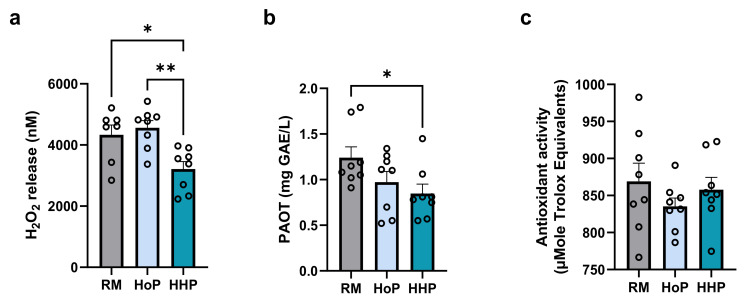
Impact of treatments by HoP or HHP processing of DM on milk H_2_O_2_ concentrations and total antioxidant capacities. (**a**) H_2_O_2_ concentration in raw (RM), HoP- and HHP-DM. Total antioxidant activity measured by PAOT-Liquid^®^ Technology (**b**) and ORAC assay (**c**). n = 8 for each group, * *p* < 0.05, ** *p* < 0.01.

HHP treatment significantly decreased both the H_2_O_2_ level and total antioxidant capacity, reported as PAOT activity, compared to raw-DM (Figure 1a,b). Conversely, HoP treatment did not impact these parameters compared to raw-DM. Finally, milk TAC assayed by ORAC assay was not affected by HoP and HHP treatment (Figure 1c).

### 3.2. Effect of a Chronic Oral Treatment of Mice with HoP- and HHP-DM on the Gene Expression Level of Some Markers of OS in the Ileum

**Figure 2:**
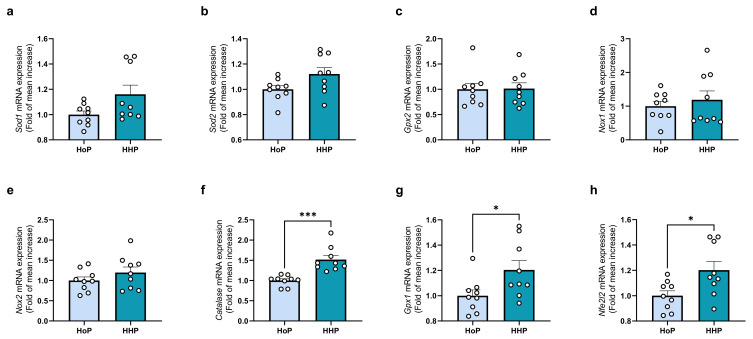
Antioxidant systems gene expression levels in ileum of mice following a 7 days gavage with HoP- or HHP-DM. (**a**) Sod1 (Superoxide dismutase 1), (**b**) Sod2 (Superoxide Dismutase 2), (**c**) Gpx2 (Glutathion peroxidase 2), (**d**) Nox1 (NADPH Oxidase 1), (**e**) Nox2 (NADPH Oxidase 2), (**f**) Catalase (Catalase), (**g**) Gpx1 (Glutathion peroxidase 1), (**h**) Nfe2l2 (Nuclear Factor Erythroid-2 like 2). n = 9 in each group, * *p* < 0.05, *** *p* < 0.001.

The quantification of the expression of genes coding for antioxidant enzymes following a 7 days gavage of mice with the different sterilized DM showed an increase in the defenses against OS. Thus, although Sod1/Sod2, Gpx2 and Nox1/Nox2 mRNA expression were unchanged (Figure 2a–e), catalase and Gpx1 mRNA, two of the main cellular antioxidant systems, were both overexpressed in mice treated with HHP-DM (Figure 2f,g). In addition, the expression of Nfe2l2 mRNA, a transcription factor regulating antioxidant proteins expression, was also increased (Figure 2h).

### 3.3. Effect of a Chronic Oral Treatment of Mice with HoP- and HHP-DM on the Gene-Expression Level of Some Markers of OS in the Liver

**Figure 3:**
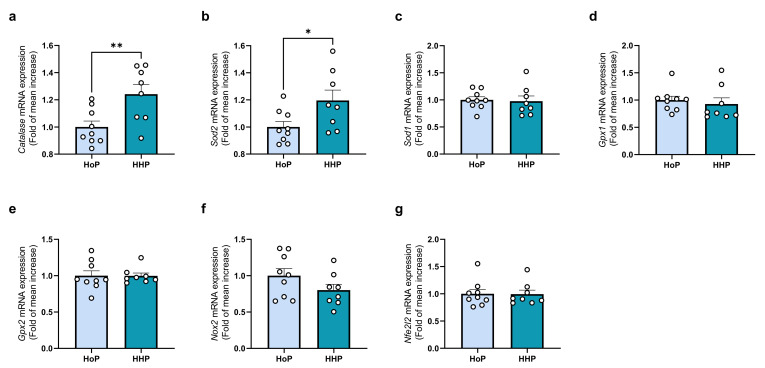
Gene expression levels of antioxidant systems in the liver of mice following a 7 days gavage with HoP- or HHP-DM. (**a**) Catalase (Catalase), (**b**) Sod2 (Superoxide Dismutase 2), (**c**) Sod1 (Superoxide dismutase 1), (**d**) Gpx1 (Glutathion peroxidase 1), (**e**) Gpx2 (Glutathion peroxidase 2), (**f**) Nox1 (NADPH Oxidase 1), (**g**) Nox2 (NADPH Oxidase 2). n = 9 in each group, * *p* < 0.05, ** *p* < 0.01.

The liver analysis of genes coding for proteins involved in the antioxidant response revealed an overall stimulation. More precisely, catalase and Sod2 mRNA expression were significantly increased (Figure 3a,b). However, Sod1, Gpx1/Gpx2 and Nfe2l2 mRNA expression remained unaffected in both groups (Figure 3c–g).

### 3.4. Effect of a Chronic Oral Treatment of Mice with HoP- and HHP-DM on the Gene-Expression Level of Some Markers of Inflammation in the Ileum and Liver

**Figure 4:**
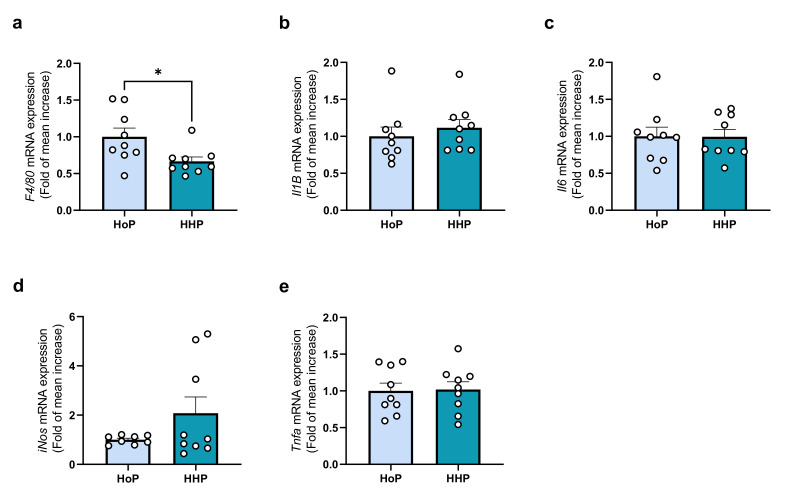
Inflammatory markers in the ileum following a 7 days gavage of mice with HoP- or HHP-DM. (**a**) F4/80 (F4/80), (**b**) Il1β (IL1β), (**c**) Il6 (IL6), (**d**) iNos (iNOS), (**e**) Tnfα (TNFα). n = 9 in each group, * *p* < 0.05.

The analysis of the gene expression level in ileum coding for proteins involved in inflammation demonstrated a decrease in F4/80 mRNA expression in the HHP group (Figure 4a). However, the other markers analyzed did not show any change between HoP and HHP groups (Figure 4b–e).

**Figure 5:**
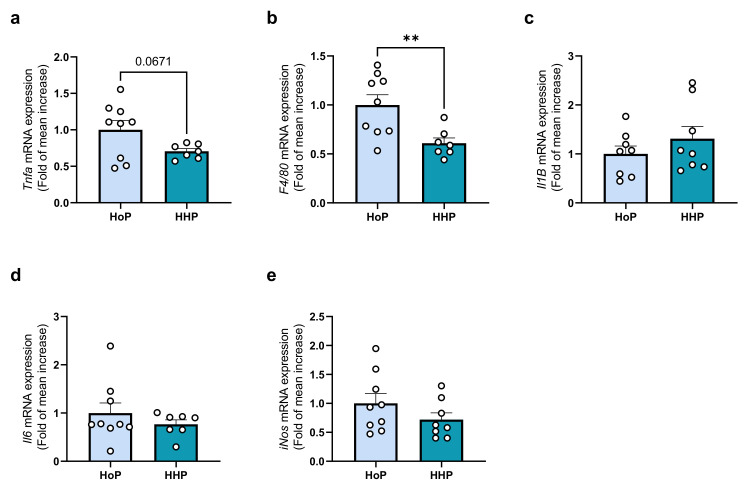
Inflammatory markers in the liver following a 7 days gavage of mice with HoP or HHP. (**a**) Tnfα (TNFα), (**b**) F4/80 (F4/80), (**c**) Il1β (IL1β), (**d**) Il6 (IL6), (**e**) iNos (iNOS). n = 9 in each group, ** *p* < 0.01.

In the liver, some markers of inflammation were decreased by HHP-DM administration, as shown by the decreased expression of Tnfα and F4/80 mRNA (Figure 5a,b). Nevertheless, Il1β, Il6 and iNos mRNA expressions were not affected in both groups (Figure 5c–e).

## 4. Discussion

In the present study, we have discovered that both HoP and HHP treatments are associated with the modification of milk antioxidants’ composition and activity. However, when administered in vivo, in adult mice, HHP-DM demonstrates a beneficial health impact on the gut and liver physiology.

Very few studies have examined the effects of HHP treatment on milk vitamins levels. Retinol (vitamin A) is involved in neurodevelopment in newborn, whose vitamin A needs are met by the high supply of this vitamin from breast milk [20]. The absence of an effect of HoP treatment on milk vitamin A observed in our study has already been reported in another study [14]. However, we show, for the first time, that HHP treatment similarly does not alter this vitamin. In the literature, conclusions on HoP and HHP effects on milk vitamin E isoforms are contradictory [14]. The use of a different method of analysis, as well as the use of different HHP protocols, might explain the discrepancies observed [14]. Here, DM treatments did not affect α-tocopherol levels. This component is the main active form of vitamin E and displays a major role as an ROS scavenger [21]. Moreover, along with two other groups, we have observed a decrease in γ-tocopherol levels in DM following HoP, demonstrating the deleterious effect of this treatment [14]. Recent studies pointed out that this component displays specific antioxidant activities as well as anti-inflammatory properties, giving it roles of equal to greater importance compared to α-tocopherol [21].

The presence of H_2_O_2_ in human milk is not well documented. It has been suggested that milk H_2_O_2_ might exert beneficial antimicrobial effects [22]. However, the impact of DM sterilization on H_2_O_2_ levels has never been tested. We found that HHP treatment decreased H_2_O_2_ levels, conversely, to HoP. Indeed, HoP treatment is known to negatively impact milk antimicrobial properties by destroying milk antimicrobial compounds, whereas HHP treatment seems to generally better preserve these effects [23]. Milk enzymes, such as catalase, participate in milk H_2_O_2_ degradation. Interestingly, Malinowska-Pańczyk et al. [24] highlighted that the milk antioxidant enzyme superoxide dismutase (SOD) activity is increased by HHP. Therefore, it could be plausible that other enzymes implicated in ROS degradation, such as catalase, may also be sensitive to HHP, thus promoting H_2_O_2_ degradation. Previous studies have shown that H_2_O_2_ found in breast milk inhibits the growth of opportunistic pathogens such as *Staphylococcus aureus* and *Salmonella* spp. [25]. It remains to be investigated if HHP-DM is likely to contribute to a better protection of preterm infants against microbial pathogens than HoP-DM.

In this study, we showed that HHP-DM displayed a reduced total antioxidant capacity (TAC) measured by PAOT-Liquid^®^ Technology, but TAC was found to be unaffected by ORAC assay. The literature is very heterogeneous concerning the effect of HoP on milk TAC, with reports of a reduction in or even absence of effects [14]. As clearly demonstrated by Sanchez-Hernandez et al. [26], these discrepancies might be due to the different methods used for TAC evaluation. Indeed, milk TAC is mostly evaluated by chemical assays, based on a single electron transfer reaction (SET). In this reaction, the redox reaction between the antioxidant and the oxidant is measured by the change in the oxidant’s color or based on a hydrogen atom transfer reaction, in which, there is a competitive reaction between the antioxidant and the substrate for the free radicals ORAC [27]. However, each method displays its own pros and cons, with different reaction media, reactions being biologically relevant or not, a lack of standardized protocol and strong environmental influence (e.g., temperature) for most methods. In addition, some reactions more likely reflect the activity of certain types of antioxidant, making a comparison between different studies complex [28]. The absence of variations in milk TAC measured by ORAC following HHP treatment is in line with another study using a Trolox equivalent antioxidant capacity (TEAC) assay [14]. For the first time, we assayed milk TAC using an electrochemical method (PAOT-Liquid^®^ Technology) and showed a significant decrease in HHP milk TAC. Thus, regarding chemical ones, we hypothesize that this method may also reflect the activity of certain types of antioxidants compared to other assays, despite this method being considered as more precise due to the use of microelectrodes to record redox potential variations [17].

Regarding the impact of HHP-DM on Nfe2l2 mRNA expression, one can speculate that the positive impact observed could be due to one (or more) milk components. In fact, we have recently published that the concentration of GLP-1 is increased in HHP milk compared to HoP milk [29]. It is well established that GLP-1 signaling could activate Nfe2l2 mRNA expression [30,31]. In addition, another possibility is that human milk may modify gut microbiota, which is known to exert an influence on the Nrfe2l2 antioxidant axis [32]. Again, in addition to the modification of GLP-1 content in milk, HHP treatment may modulate the gut microbiota composition, which is known to modulate the intestinal GLP-1 release [33]. These scientific leads need to be studied soon to explain the antioxidant action of HHP milk.

So far, clinical investigations on oxidative-stress-related outcomes for preterm infants receiving DM treated with different pasteurization methods are lacking. To draw conclusions on the clinical relevance of HoP- and HHP-DM on damage from oxidative stress, the second aim of this study was to measure some markers of OS in both the ileum and the liver of mice that were submitted to a chronic daily oral gavage with DM. We observed that HHP-DM induces an overall stimulation of antioxidant defenses in both the ileum and liver compared to HoP-DM. Furthermore, this rise in antioxidant defenses is also paralleled by a lower level of some inflammatory markers in these tissues. Of course, this preliminary study presents limitations since (1) the experiments have been performed in adult mice and not in newborn mice, and (2) the inflammation process is measured by an indirect method. Therefore, it is expected that the HHP processing of DM would benefit preterm infants by diminishing ROS-associated inflammation in the ileum and liver. Further experimental (in developing rodents) and clinical studies are therefore needed to conclude on this hypothesis.

## 5. Conclusions

In conclusion, we demonstrate that the HHP treatment of DM preserved vitamin A and E close to the raw milk level and reduced the H_2_O_2_ concentration. Our preliminary study in adult mice suggests that HHP-DM stimulates antioxidant defenses and reduces inflammation in both the ileum and liver compared to HoP-DM. This study suggests that the HHP treatment of DM may optimize the early-life nutrition and health of preterm infants.

## Figures and Tables

**Table 1:** Primers sequences.

Targeted Gene (Accession Number)	Forward Primer	Reverse Primer
*Catalase (NM_009804.2)*	TGAGAAGCCTAAGAACGCAATTC	CCCTTCGCAGCCATGTG
*Sod1 (NM_011434.2)*	GTGATTGGGATTGCGCAGTA	TGGTTTGAGGGTAGCAGATGAGT
*Sod2 (NM_013671.3)*	TTAACGCGCAGATCATGCA	GGTGGCGTTGAGATTGTTCA
*Gpx1 (NM_001329528.1)*	ATCAGTTCGGACACCAGGAGA	GTAAAGAGCGGGTGAGCCTTCT
*Gpx2 (NM_030677.2)*	TTCCCTTGCAACCAGTTCGGA	AGGATGCTCGTTCTGCCCATT
*Nox1 (NM_172203.2)*	TGCAGGCATCCTCATTTTGCG	TGGGTGCATGACAACCTTGG
*Nox2 (NM_007807.5)*	GCCAGTGTGTCGAAATCTGCT	AATTGTGTGGATGGCGGTGT
*Nfe2l2 (NM_001399226.1)*	GGTTGCCCACATTCCCAAACA	ATATCCAGGGCAAGCGACTCA
*Tnfα (NM_013693.3)*	GGGACAGTGACCTGGACTGT	TTCGGAAAGCCCATTTGAGT
*Il1β (NM_008361.4)*	ACCTTCCAGGATGAGGACATGAG	CATCCCATGAGTCACAGAGGATG
*Il6 (DQ788722.1)*	GCCCACCAAGAACGATAGTCA	CAAGAAGGCAACTGGATGGAA
*F4/80 (NM_001355722.1)*	TGACAACCAGACGGCTTGTG	GCAGGCGAGGAAAAGATAGTGT
*iNos (NM_001313922.1)*	CTCCACAAGCTGGCTCGCTT	TTCAAGCACCTCCAGGAACGT

**Table 2:** Concentrations of some antioxidant vitamins in raw-, Holder (HoP)- and High Hydrostatic Pressure (HHP) pasteurized -donor milk (DM). Data are presented as mean ± SEM. Asterisks correspond to level of statistical significance for paired comparisons with RM. * *p* < 0.05.

Antioxidant Compounds	Raw DM			HoP-DM				HHP-DM			
	Mean ± SEM			Mean ± SEM			% RM	Mean ± SEM			% RM
Vitamin A (mg/L)	0.32	±	0.04	0.29	±	0.03	−8	0.30	±	0.04	−7
α-tocopherol (mg/L)	8.75	±	0.80	8.79	±	0.98	0	9.17	±	1.60	5
γ-tocopherol (mg/L)	0.80	±	0.07	0.70	±	0.06	−12 *	0.76	±	0.07	−5

## Data Availability

Data is contained within the article.
